# Determining the efficacy of corneal crosslinking in progressive keratoconus

**DOI:** 10.12669/pjms.332.11690

**Published:** 2017

**Authors:** Sidra Malik, Sadia Humayun, Shahzad Nayyar, Mazhar Ishaq

**Affiliations:** 1 Dr. Sidra Malik, FCPS. Armed Forces Institute of Ophthalmology, National University of Medical Sciences (NUMS), Rawalpindi, Pakistan; 2 Dr. Sadia Humayun, MCPS, FCPS, FRCS. Armed Forces Institute of Ophthalmology, National University of Medical Sciences (NUMS), Rawalpindi, Pakistan; 3 Dr. Shahzad Nayyar, FCPS ENT Dept., CMH Rawalpindi, National University of Medical Sciences (NUMS), Rawalpindi, Pakistan; 4 Dr. Mazhar Ishaq, FCPS, FRCS, FRCOphth. Armed Forces Institute of Ophthalmology, National University of Medical Sciences (NUMS), Rawalpindi, Pakistan

**Keywords:** Corneal Crosslinking, Efficacy, Keratoconus

## Abstract

**Objective:**

To determine the Efficacy of Corneal Crosslinkage (CXL), using Corneal Topography, in eyes with progressive Keratoconus.

**Methods:**

This randomized control trial was conducted at Armed Forces Institute of Ophthalmology, Rawalpindi, Pakistan from October 2013 to April 2014. A total of 60 eyes of 30 patients were included who presented with bilateral progressive Keratoconus. Each eye of the patient was randomized either to a treatment group (Group-A) or control untreated group (Group-B) of 30 eyes each. A written informed consent was obtained from each patient, following which corneal crosslinkage (CXL) with topical riboflavin eye drops was performed. Follow up visit was done at three months post operatively, Corneal topography was repeated and recorded.

**Results:**

The mean age of the patients was 23.13±7.62 years (range 13 to 39 years). There were 26 males and 34 females patients. The mean simK value at the start of study was 50.94±4.84 diopters in Group-A and 49.73±5.24 diopters in Group-B. At three months follow-up, the mean simK value was significantly lower in Group-A (48.28±4.47) as compared to Group-B (51.11±4.85). Keratoconus improved/ remained stable in 34 (56.7%) eyes while progressive disease was noted in 26 (43.3%) eyes. When compared between the groups, the frequency of efficacy was significantly higher in Group-A (86.7% vs. 26.7%; p=.000) as compared to Group-B.

**Conclusion:**

Corneal Crosslinking was found effective in causing regression or halting the progression of disease in patients with progressive Keratoconus at three months follow-up, however, the efficacy of corneal crosslinking was unaffected by patient’s age and gender.

## INTRODUCTION

Keratoconus is disease of the younger age group in which progressive corneal thinning occurs and cornea assumes a conical shape associated with abnormal curvature. This change often results in irregular astigmatism and myopia and leads to mild to marked visual impairment.^1^

Corneal topography is a superior diagnostic modality in diagnosing keratoconus as it is non-invasive and can detect early stages of Keratoconus enabling timely intervention and can also aid in monitoring treatment progression.^2^,^3^

Until recently most Keratoconus treatments focused on correcting the refractive errors induced by the corneal deformation or changing the corneal geometry as in corneal transplantation, but the only treatment that aims to stop or decrease progression of Keratoconus is Corneal crosslinking (CXL).^4^ It is a procedure that uses Ultraviolet A (UVA) light in conjunction with riboflavin as a photo mediator to add additional polymer bands between collagen fibers thereby increasing corneal biomechanical resistance.^5^,^6^ It is used nowadays for stabilization of corneal tissue, which is accompanied by a reduction in the simulated keratometry (simK) values and a modest improvement in visual acuity. A study conducted by Derakhshan et al. showed that maximum and mean simK values decreased by 0.65 D and 0.51 D respectively (P < 0.05) in 77% of the treated eyes. These results were observed at three months and remained stable up to six months.^7^ Another study by Agarwal et al. also showed that maximum K value decreased by a mean of 2.47D in 54% and remained stable in 38% of treated eyes.^8^ Over the past few years, CXL has been used in various corneal disorders other than keratoconus such as non-healing corneal ulcer, PBK and Fuchs endothelial dystrophy.^9^,^10^ However, there is no established evidence of effectiveness of CXL in these conditions. Some authors claim its benefit in non-healing ulcer and symptomatic PBK while others consider this treatment ineffective.^11^,^12^

The rationale of conducting this study is to demonstrate the efficacy of CXL in progressive Keratoconus at three months following the procedure, using Galilie G4 (corneal topography). Since it is a newer, less invasive and less time consuming treatment modality it will help us treat the patients of Keratoconus more effectively who otherwise had little treatment options available.

## METHODS

This was a randomized controlled trial conducted at Armed Forces Institute of Ophthalmology (AFIO), Rawalpindi over 6 months from 23 October, 2013 to 22 April, 2014. During this period after seeking permission from hospital ethical committee, a total of 30 patients with a diagnosis of bilateral progressive keratoconus fulfilling the inclusion criteria were selected through non-probability consecutive sampling. Informed written consent was taken from all the patients. Each eye of the patient was randomized either to a treatment group (group A) or control untreated group (group B) of 30 eyes each, with the control group crossing over to the treatment group at the three month visit. Detailed history and ophthalmic examination was done and corneal topography was carried out of both the eyes before the surgery and three months following the procedure. simK values (Corneal topography) were recorded on predesigned proforma before and after surgery to check for the efficacy of CXL which was the final outcome. CXL was performed on selected eyes with isotonic riboflavin under standard protocol.

### Procedure

All the patients underwent corneal topography (Galilae G4) before surgery. CXL was performed as a day care procedure under sterile conditions. After instilling topical anesthesia, central corneal epithelium was removed with the help of sterile blunt spatula followed by instillation of riboflavin eye drops (one drop every two minutes) for 30 minutes. UVA radiations were delivered using (Vario, CCL-365) for 30 minutes at power settings of 3mW/cm2 (Standard Dresden Protocol). Bandage contact lens was placed at the end of procedure and patients were advised with topical moxifloxacin, nepafenac and cyclopentolate eye drops three times a day for 1 week. Patients were reviewed at day one, after two weeks, four weeks and then three months. Corneal topography was repeated at three month visit.

### Statistical Analysis

All the collected data was entered into SPSS version 10. Numerical variables i-e age, pre and post-op simK values have been presented by mean± SD t-test has been applied for comparison of simK values in treated versus untreated eyes at start and 3 month follow up taking p≤0.05 as significant. Categorical variables like gender, proportion of eyes showing efficacy of procedure (improvement or stability) have been presented by frequency and percentage. Chi-square has been applied taking for comparison of efficacy in treated versus untreated eyes taking p≤0.05 as statistically significant. Efficacy has been stratified for age and gender to address effect modifiers. Post-stratification chi-square test has been applied taking p≤0.05 as statistically significant.

## RESULTS

This study involved 60 patients with a mean age of 23.13±7.62 years, amongst these 30 patients underwent surgery. There were 26 males and 34 females. The mean simK value at the start of study was 50.94±4.84 diopters in Group-A and 49.73±5.24 diopters in Group-B. At three months follow-up, the mean simK value was significantly lower in Group-A (48.28±4.47) as compared to Group-B (51.11±4.85) as shown in Table-I(a). Keratoconus improved/remained stable in 34 (56.7%) eyes while progressive disease was noted in 26 (43.3%) eyes and when compared between the groups, the frequency of efficacy was significantly higher in Group-A (86.7%) as compared to Group-B (26.7%) as shown in Table-I(b).

**Table-I (a):** Comparison of Mean simK values in the Two Groups at 3 Months Follow-up

	*Study Groups*		*P value*

	*Group-A (Eyes with CXL)*	*Group-B (Eyes without CXL)*
simK (diopters)	48.28±4.47	51.11±4.85	.022

**Table-I (b):** Comparison of Efficacy between the Study Groups

			*Study Groups*		*Total*	*P value*

			*Eyes with CXL*	*Eyes without CXL*
Efficacy	Not effective	Count	4	22	26	0.000
		% within Group	13.3%	73.3%	43.3%
	Effective	Count	26	8	34
		% within Group	86.7%	26.7%	56.7%
	Total	Count	30	30	60	
		% within Group	100.0%	100.0%	100.0%	

## DISCUSSION

The only proven treatment that halts or decreases progression of Keratoconus is Corneal crosslinking (CXL).^4^,^8^,^9^ CXL is a novel treatment which is not only less invasive but is also less time consuming treatment modality which was introduced in our region and within a span of few years has become very prevalent and choice modality in progressive keratoconus. Apart from other benefits, CXL sometimes delays or even does away with the need of corneal transplantation in these patients.

CXL has been tried in the management of patients with progressive keratoconus to halt the disease progression with fruitful results,^2^,^7^,^13^-^15^ the efficacy of which varies between various studies from as low as 40.91% in UK^11^ to as high as 98% in Germany.^2^ A possible explanation for this conflict can be the differences in the prevalence and pattern of keratoconus among various populations. United Kingdom based studies have revealed rates of keratoconus in people of Asian descent (most commonly from Northern Pakistan) born in the UK to be as high as one in 4000 per year, compared with only one in 30 000 per year for local white Caucasian populations. These data suggest that ethnic background significantly influences the incidence and the severity of keratoconus, since the Asian subjects present at a significantly younger age than Caucasian subjects and also appear to suffer from a more severe form of the ectatic disease.^16^,^17^

Due to this conflict among exiting literature, population and geographic differences and lack of local research, the purpose of the current study was to demonstrate the efficacy of CXL in treatment of eyes with progressive Keratoconus at three months following the procedure using Galilie G4 (corneal topography) in local population. Our results match with those of Sharma et al.^15^ in 2015 (India) who observed efficacy of CXL to be 91% in 32 eyes with progressive keratoconus and Raiskup-Wolf et al.^2^ in 2008 (Germany) who observed the efficacy of CXL to be 98% in 241 eyes. In our study the efficacy of corneal crosslinking was unaffected by patient’s age and gender.

A very important limitation of the current study is short-term follow-up. Also in the present study complications of CXL like secondary infection, temporary corneal haze, permanent scars, endothelial damage, sterile infiltrates, and herpes reactivation were not considered. Therefore studies with a larger cohort and long term follow up are recommended to confirm the efficacy and safety of CXL in the management of progressive keratoconus.

## CONCLUSION

In our study CXL was found to be effective in causing regression or halting the progression in patients with progressive Keratoconus at three months follow-up, however, the efficacy of corneal crosslinking was unaffected by the patient’s age and gender.

### Authors’ Contribution

**SM:** Conceived, designed and did statistical analysis & editing of manuscript.

**SH and SN:** Did data collection and manuscript writing.

**MI** did review and final approval of manuscript.
